# Mycotherapy: Potential of Fungal Bioactives for the Treatment of Mental Health Disorders and Morbidities of Chronic Pain

**DOI:** 10.3390/jof8030290

**Published:** 2022-03-11

**Authors:** Elaine Meade, Sarah Hehir, Neil Rowan, Mary Garvey

**Affiliations:** 1 Department of Life Science, Sligo Institute of Technology, F91 YW50 Sligo, Ireland; elaine.meade@mail.itsligo.ie (E.M.); hehir.sarah@itsligo.ie (S.H.); 2 Centre for Precision Engineering, Materials and Manufacturing Research (PEM), Institute of Technology, F91 YW50 Sligo, Ireland; 3 Bioscience Research Institute, Technical University Shannon Midlands Midwest, N37 HD68 Athlone, Ireland; neil.rowan@tus.ie

**Keywords:** fungi, biologics, mushrooms, chronic pain, mycotherapy, mental health

## Abstract

Mushrooms have been used as traditional medicine for millennia, fungi are the main natural source of psychedelic compounds. There is now increasing interest in using fungal active compounds such as psychedelics for alleviating symptoms of mental health disorders including major depressive disorder, anxiety, and addiction. The anxiolytic, antidepressant and anti-addictive effect of these compounds has raised awareness stimulating neuropharmacological investigations. Micro-dosing or acute dosing with psychedelics including Lysergic acid diethylamide (LSD) and psilocybin may offer patients treatment options which are unmet by current therapeutic options. Studies suggest that either dosing regimen produces a rapid and long-lasting effect on the patient post administration with a good safety profile. Psychedelics can also modulate immune systems including pro-inflammatory cytokines suggesting a potential in the treatment of auto-immune and other chronic pain conditions. This literature review aims to explore recent evidence relating to the application of fungal bioactives in treating chronic mental health and chronic pain morbidities.

## 1. Introduction

There are many recognised mental health disorders or mental illnesses including (but not limited to) anxiety disorders, mood disorders (depression, bipolar disorder, and cyclothymic disorder) psychotic disorders (schizophrenia), eating disorders (anorexia nervosa, bulimia nervosa), impulse control and addiction disorders, obsessive-compulsive disorder (OCD), post-traumatic stress disorder (PTSD) and personality disorders [1]. Each mental illness manifests with a variable array of symptoms that differ depending on the illness present; it is accepted, however, that a person’s mood, thinking, perceptions, anhedonia (pleasure sensation) and behaviours are affected. According to the 2013 Diagnostic and Statistical Manual of Mental Disorders 5 (DSM-5), “A mental disorder is a syndrome characterized by clinically significant disturbance in an individual’s cognition, emotion regulation, or behaviour that reflects a dysfunction in the psychological, biological, or developmental processes underlying mental functioning” [1]. These issues are undoubtedly proliferated by the stigma toward mental disorders such as anxiety, clinical depression (major depressive disorder), and OCD where a lack of understanding in society prohibits afflicted persons from seeking help or speaking out [2]. Stigma where mentally ill persons are perceived as dangerous, unpredictable, or as having a weakness of character has excluded them from society to an extent, further exasperating the issue [2]. The use of the DSM-5 in the diagnosis of mental illness, however, remains under scrutiny as understanding and expansion of mental illness progresses [3]. The World Health Organisation (WHO, Geneva, Switzerland) created the WHO International Consortium in Psychiatric Epidemiology (ICPE) in 1998 with the aim of gathering data on mental illness via a diagnostic instrument, i.e., the WHO Composite International Diagnostic Interview (CIDI) surveys [4]. The WHO states that mental health illness is increasing yearly across the globe with approximately 20% of children and adolescents being diagnosed with a mental illness where suicide is the second leading cause of death in 15- to 29-year-olds [5]. In Europe, mental health disorders affect approximately 165 million people yearly with anxiety, mood and addictive disorders being most common [6]. Suffering a mental health disorder is devasting for the individual afflicted but also impacts the family unit as substance abuse and suicidal idealisation often manifests. Considering the wider burden of mental health disease and its associated functional impairment, the socio-economic impact must also be considered due to their increasing prevalence and high disability rate where treatment options often remain unmet [7].

The direct economic burden mental illness relates to diagnosis, treatment, or hospitalisation costs with indirect costs associated with the impact on economic growth including loss of income due to disability, absence from work and cost of additional care [6].

The impact of chronic pain co-morbidities associated with mental health must also be considered including functional disorders such as fibromyalgia and other functional somatic syndromes (FSSs). The treatment of mental health disorders is typically of combination of drug therapy based on psychoactive drugs (Table 1), mood stabilisers or antipsychotic drugs, and psychotherapeutic treatments. In many cases, however, mentally ill persons are non-responsive to therapy, termed treatment resistant [8]. Currently, 30% of clinically depressed are treatment resistant with this cohort having increased issues with social interactions, occupational difficulties, declining physical health and often suicidal thoughts [9]. Additionally, therapeutics currently in use are prone to numerous side effects where treatment is ultimately discontinued in patients (Table 1). To provide therapeutic options for such persons, and to reduce the burden of disease, it is imperative that we seek effective alternatives. The use of psychedelics, such as psilocybin (magic mushrooms), Lysergic acid diethylamide (LSD), and ayahuasca have drawn attention due to their noticeable ability to alter consciousness in personally meaningful, therapeutic, and spiritual ways [10]. Indeed, clinical trials on psilocybin demonstrated its efficacy in reducing cancer-related anxiety and depression, treatment-resistant depression, major depressive disorder (MDD), and substance misuse [11]. This review aims to highlight and discuss the potential of fungal biologics including psilocybin, LSD and others as therapeutic alternatives (mycotherapy) for alleviating the symptoms of mental health disorders. The current knowledge underlining the potential of fungal biologics is highlighted and discussed in the context of common mental health disorders including addiction, MDD and anxiety where current treatment is ineffective or causes significant undesirable side effects. Additionally, this review will review the potential of said biologics to also alleviate the symptoms of chronic pain conditions commonly associated with mental illness including fibromyalgia and irritable bowel syndrome.

## 2. Mental Health Disorders

The incidence of mental health disorders is increasing globally, yet a fundamental understanding of the aetiology of mental health disease remains elusive. Assumptions relate severe mental illness to a small number of genes with a specific relationship between genotype and mental illness where biological pathways and environmental factors impact on illness [43]. Studies have demonstrated a strong relationship between severe trauma such as childhood abuse and/or abandonment with adult mental health disorders and a diminished quality of life [44]. The most widely discussed theories of depression are based on the regulation of the monoamines, norepinephrine (NE), dopamine and serotonin. The catecholamine hypothesis of depression developed in the 1960s remains the basis of treatment of mental health disorders such as MDD and anxiety disorder. Based on the theory that such mental illness is a result of a NE or dopamine deficiency at central nervous system (CNS) synapses, treatment aims to increase dopamine or NE levels to alleviate symptoms [45]. The serotonin hypothesis of depression also from the 1960s relates mood disorders to a deficiency in serotonin (5-hydroxytryptamine, 5-HT) a neurotransmitter involved in regulating emotion and mood [46]. There are currently 14 5-HT receptor subtypes identified having varying affinities for serotonin, present presynaptically, postsynaptically in the CNS and throughout the brain being predominately G protein coupled receptors (except 5-HT3 receptor) [47]. Agonism of the 5-HT_2A_ receptors is involved in increasing cortical glutamate levels [48]. The neuroendocrine hypothalamic-pituitary-adrenal (HPA) axis a natural pathway activated in times of stress also becomes hyperactive in states of depression. The HPA axis is involved in maintaining blood glucocorticoid levels, where elevated levels can disrupt HPA function [49]. Studies have shown that cortisol elevation long term results in cognitive and other medical issues in patients and exacerbate depressive symptoms as shown in mice [50]. Thus, it is generally accepted that neuropsychiatric diseases such as PTSD, MDD, anxiety etc are a result of a chemical imbalance in neural circuits distributed across the brain and CNS related to a loss of neuro plasticity [51]. Consequently, treatment of mental health disorders is based on prescribing anti-depressants namely selective serotonin reuptake inhibitors (SSRIs), serotonin-norepinephrine reuptake inhibitors (SNRIs), dopamine reuptake inhibitors (DRIs) and tricyclic antidepressants (TCAs) which aim to increase neurotransmitter levels at the synapses and increase neuroplasticity. While the efficacy of this approach remains under question the side effects associated with the varying therapeutics in these categories are undeniable. Serotonin syndrome for example which is a potentially life-threatening condition can be caused by using serotonergic drugs and overactivation of 5-HT_1A_ and 5-HT_2A_ receptors [52]. Additionally, antidepressants such as TCAs and SSRIs are known to increase the concentration of neurotransmitters at synapses within minutes, yet it takes weeks to months for mental illness symptoms to regress in patients [53]. Additional theories of depression include the neurogenic hypothesis of depression and inflammatory hypothesis based on an increase in proinflammatory cytokines in the nervous system [7]. The 5-HT receptors are known to regulate the release of cytokines interleukin (IL) and tumour necrosis factor (TNF) and modulate immune macrophage and dendritic cell function and aid in maintaining an anti-inflammatory state in vivo [54]. With increasing rates of mental illness and suicide ideation and tendencies, it is imperative that a more rapid acting treatment option is established.

### Mental Health and Co-Morbidities of Chronic Pain

Functional somatic syndrome is the term used for conditions that do not appear to have an underlying biological pathology [55]. These debilitating conditions are becoming increasingly frequent imposing a significant economic burden. Chronic fatigue syndrome (CFS), irritable bowel syndrome (IBS) and fibromyalgia (FM) amongst others are FSSs where pain, fatigue, muscle, and joint aches often present without an identifiable cause. Theories have arisen (the ‘Lumpers’ vs. ‘Splitters’ debate) suggesting that certain FSSs have one underlying aetiology and are associated with psychiatric disorder’s including anxiety and depression. While the pathophysiology of FM remains unknown it has been postulated that thinly myelinated and unmyelinated C-nerve fibres have been reported in FMS patients [56] suggesting a link between loss of nerve myelin and chronic pain. Additionally, studies suggest that a dysfunction of dopamine is present in FM patients where dopamine agonists appear effective in treating symptoms [57]. Studies have demonstrated decreased cortical dopamine receptor binding in FM patients [57]. FM characterised by extreme hyperalgesia is highly prevalent (3 times more prevalent) in persons with major depressive disorder than in patients without, studies also show high rates of MDD amongst family members of FM patients suggesting a genetic aspect [58]. Additionally, sleep abnormalities in FM are similar to those present with a 5-HT dysfunction in depressed individuals [58]. CFS appears less linked to mental health disorders where psychiatric status is not considered an important causal contributor to CFS [59]. IBS is a painful syndrome characterized by chronic abdominal pain, altered bowel habits, correlated to significant psychological distress and psychiatric comorbidities anxiety, panic disorder, MDD and suicidal ideation [60]. Polymorphisms in the serotonin transporter gene (SERT) has been associated with higher risk of depression in IBS patients with a dysfunction of serotonin transporters impacting the emotion regulating regions of the brain [61,62]. At present, treatment of FM and IBS is also reliant on the use of antidepressant drugs TCAs and SNRIs where successful therapy has supported the belief that a dysfunction of serotonin and NE is present in FSSs similarly to MDD [58].

The relationship between mental health disorders and autoimmune conditions (including multiple sclerosis, rheumatoid arthritis, colitis, and lupus erythematosus) has been well established where there is increased risk of MDD, anxiety and schizophrenia in autoimmune patients. Theories suggest the excessive inflammation characteristic of autoimmune diseases may be a casual factor impacting the CNS or via cytokine interactions between nerve and immune cells of the patient [54]. The relationship between increased levels of cytokines including tumour necrosis factor-α (TNF-α) and IL-6 and major depression disorder is now recognised [47]. This raises the question if fungal extracts showing promise in treating mental health disorders may also alleviate the symptoms of certain FSSs namely FM and IBS and auto immune conditions.

## 3. Fungal Biologics: Unlocking the Potential of Eastern Practice into Western Medicine

There has been an increasing interest in translating potential medicinal cures derived from medicinal mushroom used for centuries in Eastern practice into Western medicine [63,64]. Modern medical practice relies heavily on the use of highly purified pharmaceutical compounds whose purity can be easily assessed and whose pharmaceutical activity and toxicity show clear structure-function relationships. In contrast, for decades, many mushroom-derived medicines contain mixtures of natural compounds had not undergone detailed chemical analyses and whose mechanism of action had not been elucidated [65]. With the decline in the number of new therapeutics produced from the pharmaceutical industry, novel biologic agents are being sought from traditional medicine. Translating traditional Eastern practices into acceptable evidence-based Western therapies is challenging given different manufacturing standards, criteria of purity, and under-powered clinical trials making assessment of efficacy and toxicity by Western standards of clinical evidence difficult. Purified bioactive compounds derived from medicinal mushrooms using appropriate drug discovery programs are a potentially important new source of psychoactive agents. For example, the therapeutic potential of medicinal mushrooms is evidenced by the fact that two glucan isolates were licensed as drugs in Japan as immune-adjuvant therapy for cancer in 1980. Moreover, Murphy et al. (2021) noted that approximately 200 registered clinical trials have appeared in the literature focusing on use of beta-glucans extracted from a diversity of medicinal mushrooms with a focus on cancer treatments using mainly oral administration [65]. These authors observed significant challenges exist to further clinical testing and translation of mushroom-derived biologics. The diverse range of conditions for which biologics from medicinal fungi are in clinical testing underlines the incomplete understanding of the diverse mechanisms of action that is a key knowledge gap. By far the greatest mechanistic information relates to elucidating immunomodulatory potential of medicinal mushrooms [66,67]. Furthermore, important differences appear to exist in the effects of apparently similar mushroom-derived preparations, which may be due to differences in sources and extraction procedures, another poorly understood issue [65]. In general, there is a dearth in evidence-based knowledge from registered clinical trials on potential use of biologics derived from medicinal mushrooms for alleviating mental health conditions. Therefore, there is growing interest in adopting a Quadruple Helix approach (academia–industry–policy–society) to support emerging industries, including medicinal fungi companies, in the ‘bioprospecting’ and development of new therapeutic products informed by compliance with appropriate regulatory framework and clinical trials; moreover, this multi-actor framework also enables greater societal awareness of the potential for higher fungi to meet a diversity of emerging needs [68,69,70].

**Figure 1:**
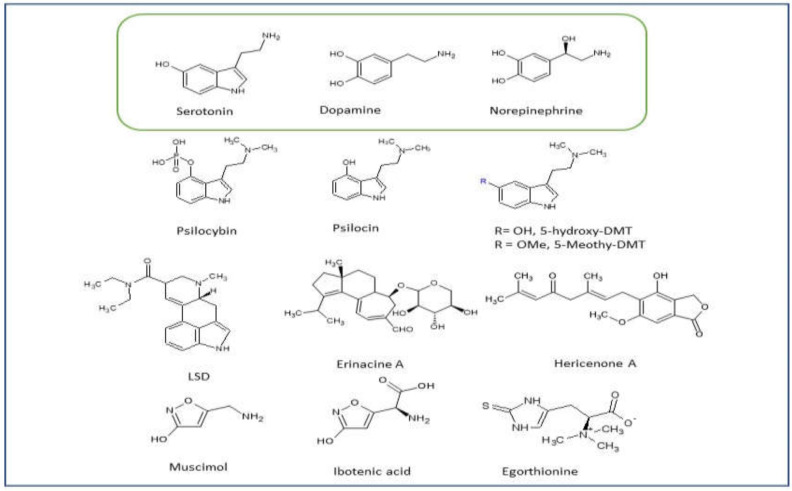
Monoamine neurotransmitters and fungal bioactives.

Outside of the use of extracts and along with the beta-glucans, a plethora of fungal bioactives have been isolated and fully purified including further polysaccharides, peptides and polyphenols, medium-sized macromolecules such as triterpenoids and the small molecule type structures of the indole alkaloids including tryptamines. Yildiz et al. (2015), for example, reports on the potent antioxidant action of phenol compounds in many medicinal mushrooms [71]. The focus here will be on the bioactives with low molecular weights which can cross the BBB and have structural features and effects similar to the common neurotransmitters. The neurotransmitters, serotonin, dopamine and NE are monoamines derived from either tyrosine or tryptophan. Some of the most interesting and potent compounds discussed herein are derived from the same precursors. Indeed, serotonin itself and psilocybin are in fact both substituted tryptamines (Figure 1).

Psychedelics or serotonergic hallucinogens are natural substances which have agonism for the 5-HT receptors and include psilocybin, dimethyltryptamine (DMT) from the chacruna (ayahuasca brew) and jurema plants and mescaline from the peyote and San Pedro cacti [72]. Psychedelic compounds produce hallucinogenic experiences via activity in brain regions regulating cognition, emotions, self-awareness and perception of pain [73]. Psychedelics are included in the psychoplastogens class of compounds which have effect on neural plasticity in key circuits involved in brain health [53]. By definition psychoplastogens produce a quantifiable change in plasticity within a short time frame (>72 h) post administration [74]. Agonism of the 5-HT receptor group is associated with neuroplastic changes potentially reducing depression and anxiety symptoms [75]. Studies show that psychoplastogens have a range of therapeutic potential in treating neuropsychiatric diseases including anxiety disorder, mood disorders, PTSD, and addiction [74]. The 5-HT receptor group also has an important role in the gut-brain axis and is linked to the pathophysiology of IBS via dysregulated serotonin signalling [76]. Mushrooms containing psychoplastogens have been in use as traditional medicine in numerous cultures as healing rituals for millennia. Fungal biologics of therapeutic potential include lysergic acid diethylamide (LSD) a synthetic derivative of the precursor LSA (d-lysergic acid) or ergine from the rye ergot fungus *Claviceps purpurea*, muscimol and a DMT derivative from *Amanita muscaria* mushroom (amongst others) and psilocybin, a hallucinogenic pro-drug of psilocin found in Psilocybe mushrooms (magic mushrooms) (Table 2). Psilocybe alkaloids are more recently obtained from magic truffles (fungis sclerotia) as this species is currently overlooked in the prohibition laws banning sale [48]. Non hallucinogenic fungal biologics showing potential for mental health treatment include compounds from the edible mushroom *Hericium erinaceus* and ergothioneine from *Pleurotus cornucopiae*.

### 3.1. Psilocybe Mushrooms

Undoubtedly psychedelics from the Psilocybe species are currently the more commonly investigated fungal compounds for their biological and therapeutic potential. Psychedelic compounds are known to exert their effects via activation of serotonergic 5-HT receptors dispersed throughout the central and peripheral nervous system impacting on cognition, mood and emotion [93]. Psilocybin is a potent agonist of 5-HT_2A_ receptors and moderate agonist of 5-HT_1A_ and 5-HT_2C3_ receptors present in the thalamus and cortex of the brain [79]. These receptors are also associated with peripheral and central nervous system pain perception [73] justifying the use of SSRIs in the treatment of chronic pain conditions such as FM. Psilocybin has also demonstrated affinity for dopamine receptors and serotonin transporter protein [94]. Both psilocybin and its metabolite psilocin, pass through the blood–brain barrier (BBB) where psilocin is 1.5 times more potent. Indeed, between the 1950s and 1970s, researchers actively investigated the potential of psilocybin in the treatment of OCD, addiction, and neurotic behaviours [72] but studies were inhibited by prohibition until the early 1990s. Psychedelics are now believed to have additional beneficial biological activities including promoting neural plasticity and immune modulation [95]. Additionally, psilocybin positively impacted symptoms of MDD, treatment resistant depression, substance addiction and anxiety (anxiolytic) in afflicted persons and terminal cancer patients [96]. Comparative studies where one dose of ca. 30 mg/70 kg psilocybin was administered to terminally ill patients reduced symptoms of depression and anxiety and improved optimism in 51 cancer patients for up to 6 months post administration [97]. In one trial study, psilocybin reduced symptoms of MDD, anxiety and anhedonia as early as 1 week post administration of 10 mg and 25 mg (seven days apart) with no observed side effects to the patient [75]. Studies assessing the impact of psilocybin on alcohol addiction showed patients had significantly fewer drinking days in the treatment period of 8 weeks at 300 µg/kg 4 weeks apart [79]. Promising results were also observed with the use of psilocybin in conjunction with cognitive behavioural therapy (CBT) to aid in smoking cessation [98]. A long term follow up study reported 60% abstinence rates after more than 12 months compared with a 31% anstinence rate at 12 months using conventional therapies. Small scale clinical studies also demonstrate the efficacy of psilocybin at reducing the symptoms of OCD where SSRI treatment failed [99]. Similar to the action of SSRIs on 5-HT receptors alleviating chronic pain in patients with neuropathic, musculoskeletal pain and fibromyalgia, psilocybin may reduce chronic pain symptoms [73]. Clinical trials are warranted on the administration of psilocybin to chronic pain patients including FSSs comparative to current treatment options such as SSRIs and opioids.

### 3.2. Claviceps purpurea

The fungus *Claviceps purpurea* is extensively known for its production of ergot alkaloids having activity on the nervous system and smooth muscles where therapeutic application includes treating Parkinson’s disease, cluster headaches and migraine [100]. LSD is a semi-synthetic derivative of lysergic acid found in fungus *Claviceps purpurea* [73]. LSD was also a psychedelic compound highly researched in the 1950s until the Controlled Substances Act was introduced in 1970 [72]. Like psilocybin, LSD has activity on brain processes involved in cognition, emotion, and perception via affinity for serotonin receptors [101]. LSD is an agonist of 5-HT with high affinity for 5-HT2A and 5-HT2C and is small enough to pass the BBB being approximately 100 times more potent than psilocybin [73]. Interestingly, LSD is also an agonist of the dopaminergic receptors with relatively high affinity for dopamine 2 receptors [102] and an agonist of the trace amine associate receptor 1 (TAAR1) [103]. Currently, in the treatment of psychotic illness the therapeutic efficacy of active pharmaceutical ingredients (APIs) is required to be greater than 70% occupancy of D2 receptors [103]. TAAR1 is essential in the regulation of monoamines (dopamine, NE, serotonin) in neurons of the CNS and neuro immune modulation [104] via agonism of trace amines (β-phenylethylamine, p-tyramine, and tryptamine). Dysregulation of trace amines and TAAR1 receptor dysfunction has been identified in psychotic disorders including MDD, bipolar disorder and schizophrenia [103]. Additionally, the TAAR1 receptors are associated with the pathophysiology of IBS [76]. Studies have demonstrated the positive effects of LSD on anxiety relating to terminal illness [93] with positive affects also observed for PTSD and addiction. The analgesic activity of LSD has also been described in terminally ill patients lasting up to 12 h post administration [73]. While reports on pain management of headaches are self-reported, patients state that sub hallucinogenic concentrations (micro dosing) of LSD prophylactically and metaphylactically alleviated migraine and cluster headaches [105]. One clinical study determined that 20 µg LSD increased pain tolerance and reduced pain perception in patients compared to placebo with similar outcomes to oxycodone and morphine [106]. Such analgesic effects may be attributed to the dopaminergic activity of LSD suggesting positive effects on neurological disease such as FM. LSD’s mechanism of action is pleiotropic affecting serotonin (5-HT), dopamine and TAAR1 receptor pathways potentially alleviating symptoms of mental illness and chronic pain conditions FM and IBS. It is important to note that LSD is a complex molecule, effecting many receptor pathways, where long-term administration may result in psychotic-like symptoms or Hallucinogen Persisting Perception Disorder (HPPD) or flashbacks in the user [103]. Such effects, however, are deemed low risk, uncommon and mostly associated with recreational use [107]. Additionally, secalonic acid A and its isomers are also found in Claviceps purpurea which has anti-cancer activity via topoisomerase I and II inhibition [108].

### 3.3. Amanita muscaria

*A. muscaria* contains many biologically active compounds including the psychoactive alkaloids: muscarine, ibotenic acid and muscimol [109] where ibotenic and muscimol are structurally similar to gamma-aminobutyric acid (GABA) having effect on glutamate receptors in the CNS [110] and can cross the BBB. Ibotenic acid is a potent neurotoxin via activation of N-methyl-d-aspartate (NMDA) receptors where muscimol has a strong psychoactivating action [111]. The alkaloid muscarine is an acetylcholine agonist of the parasympathetic nervous system typically negatively impacting the functioning of numerous organs but cannot pass the BBB [109]. Over stimulation of muscarinic cholinergic system and acetylcholine is associated with the aetiology of depression suggesting that the agonist muscarine will elevate depressive symptoms in patients. Studies by Corbett 1991, demonstrate that muscimol improved social behaviour and had anxiolytic-like effects post systemic administration to test rats, similar to diazepam [112]. Additionally, muscimol when used as a combination therapy with endomorphin-1 reduced the symptoms of neuropathic pain due to spinal cord injury when administered for 7 consecutive days [113]. Bufotenine (5-HO-DMT) is an alkaloid and a substituted tryptamine similar to serotonin and psilocin, also present in the Amanita mushroom. As such, it has the ability to cross the BBB having agonistic effects on 5-HT1A and 5-HT1B receptors [114]. Another substituted tryptamine,5-MeO-DMT, requires enzymatic activity of a monoamine oxidase (MAO) inhibitor to induce its psychedelic effects orally [48]. Recently, β-carbolines, which are well-known MAO inhibitors have been isolated directly from psilocybe mushrooms. β-carbolines, as well as psilocybin itself, are derivatives of tryptophan. This represents an interesting natural product pathway where two different types of products from the same precursor contribute to the same pharmacological effect [115]. Tryptamines have been used to treat symptoms of PTSD, MDD, addiction and anxiety [116] and show potential as treatment with low risks for addiction.

### 3.4. Hericium erinaceus (H. erinaceus)

The medical benefits of *H. erinaceus* have been well documented with anticancer, antioxidative, anti-inflammatory and antimicrobial activity amongst others [7] where neurotropic compounds (Figure 1) present in the mushroom are more recently being considered for neuropsychiatric disorders. Hericenones and erinacines (cyathane derivatives) are biological neurotropic compounds found in the fruiting body and mycelium of *H. erinaceus*. These compounds can pass the BBB due to their low molecular weight [117] which may beneficially impact on Alzheimer’s and Parkinson’s disease due to their impact on nerve growth factor (NGF) [118]. Indeed, many biological active compounds are present in the fruiting bodies of *H. erinaceus* including aromatic compounds, diterpenoids, steroids, and polysaccharides [119]. NGF is essential for protecting nerve tissue and maintaining neuron functionality where studies have shown NGF levels in MDD patients is also significantly reduced [120]. Interestingly, NGF itself is unable to pass the BBB highlighting the benefits of these compounds which stimulate NGF production within the brain and encourage nerve myelination throughout the CNS [121]. Perhaps such myelination effects may also aid in alleviating the symptoms of FM in patients where improper myelination of nerves is a possible issue [56]. Initial studies suggest that the NGF enhancing activity of these extracts may reduce MDD and anxiety [122]. Elevated levels of NGF are associated with neuroplasticity and neurogenesis which may improve mood in MDD patients based on the neurogenic hypothesis of depression [7]. Additionally, in vivo studies on depressed animals demonstrated that when fed with *H. erinaceus* levels of the monoamine’s serotonin, dopamine and NE were elevated [123]. In vivo studies report that erinacine can increase monamine expression and modulate anti-inflammatory brain-derived neurotrophic factor (BDNF) signalling in depressed animals [124]. Amycenone, a proprietary mixture of compounds which are present in the fruiting body of *H. erinaceus*, demonstrated anti-inflammatory activity against cytokines TNF and IL and reduced inflammatory induced depression in test animals [119] a similar activity observed with SSRIs and SNRIs. Such anti-inflammatory activity may also prove beneficial in alleviating symptoms of autoimmune co-morbidities of mental illness. Studies show when consuming H. erinaceus adults with mild cognitive impairment and menopausal women were less depressed, anxious with improved cognitive abilities compared to placebo control groups [121]. Studies are warranted however, confirming the efficacy of biological extracts from *H. erinaceus* mushrooms comparative to consuming the entire fruiting body of the mushroom at a therapeutic level. Additionally, while these compounds have been shown to affect the levels of monoamines, the mechanism of modulation requires further investigation.

### 3.5. Pleurotus cornucopiae

One of the fungal bioactives with the most diverse effects and potential uses currently under widespread investigation is Ergothionine. Found in large amounts in oyster mushrooms (*Pleurotus cornucopiae* var. *citrinopileatus*), Ergothione is a metabolite of the aromatic amino acid histidine. It has been shown to have neuroprotective and anti-inflammatory effects as well as being potentially involved in neurogenesis, all of which are characteristics of compounds which may be useful in the treatment of the aforementioned disorders [125]. Dietary ergothionine was shown to significantly reduce immobility time in forced swim test (FST) and tail spin test (TST) which are used as models of depression in mice [126]. Additionally, oral administration of ergothioneine prior to and during another mouse model of depression (Social Defeat Stress (SDS) paradigm) had a preventative effect on depressive behaviours such as social avoidance and sleep abnormalities [127]. Ergothioneine is a substrate of carnitine/organic cation transporter OCTN1 and this has led to investigation of the potential targeting of such atypical monoamine transporters as a strategy for the development of new anti-depressants [128].

## 4. Pharmacological Consideration of Mycotherapy

Bioactive compounds which promote neuro plasticity and induce long term changes in mood, emotion and cognition may offer therapeutic options to chronically mentally ill patients [95]. When considering the administration of psychedelic compounds as therapeutics the efficacy of single dosing versus micro-dosing must be considered as current studies vary between self-medicating/micro dosing patients and single dose clinical trials. The time frame required for onset of action is an important consideration as current drug therapy (SSRI, NSRIs) for mental illness requires weeks to months for therapeutic effect. Studies indicate the effects of the fungal bioactives described occur rapidly and have a prolonged lasting effect on the symptoms of the diseases investigated [93]. Additionally, there appears to be no, or limited side effects or withdrawal symptoms associated with acute therapy or micro-dosing. Tolerance can develop however, relating to down regulation or de-sensitization of the 5-HT receptors and a cross tolerance with other psychedelic compounds may occur [47]. Psilocybin has low toxicity with chronic exposure and moderate toxicity in cases of acute exposure and has low addiction and dependence issues [75]. LSD which is more potent than psilocybin induced some dose dependent physical and psychological symptoms including derealization, depersonalization and dissociation [106]. Psilocybin has pharmacodynamic effect lasting between 4–6 h with LSD lasting up to 12 h, an elimination half-life of 3.5 h and 3 h for LSD [129] and psilocybin [130] respectively, has been established. Route of administration is an important formulation consideration for psychotherapy as tryptamine and its derivates are prone to first pass metabolism. The addition of a monoamine oxidase inhibitor may be needed for oral delivery formulations [48]. Such an inhibitor would increase bioavailability and pharmacological efficacy of bioactive amines including psilocybin and LSD. The administration of such compounds is not straight forward as each has specific receptor affinities, duration of action and potency. The therapeutic index for each compound must be established to fully determine the safety profile as long-term treatment may be required for chronic mental health illness or chronic pain conditions. Currently, mood and behavioural studies are conducted in animals where analysis of symptoms and extrapolation to humans is difficult. Consuming such compounds may affect the patient’s consciousness and provide insights and existential and spiritual questions [99]. For example, at 12 month follow up in the smoking cessation work by Johnson et al., 86.7% of the participants rated their psilocybin experience among the 5 most personally meaningful and spiritually significant of their lives. Administration of these compounds, therefore, must be in a controlled manner to avoid the risk to persons prone to psychosis and to determine the patient’s mindset prior to administration. To this end, many of the studies involving the psychedelics and compounds which alter consciousness have been carried out in the presence of psychiatrists and counselling professionals specifically trained in psychedelic psychotherapy. Some alternative methods of determining activity have been utilized based on gene ontology, predictive analysis and Kyoto Encyclopedia of Genes and Genomes (KEGG) pathway analysis to investigate fungal biologics [124].

There is a pressing need to reach international consensus and agreement on the defined production methods for bioactives produced from medicinal fungi for this potential purpose as batch-to-batch variation in production methods and types of mushrooms may generated different structural-bioactives that may lead to vary functional activities [67,131,132]. This situation will be addressed by promoting international collaborations between academia and industry partners in the areas of biotechnology, mycology, toxicology, bioinformatics and biopharma.

## 5. Conclusions

Bioactives found in higher fungi such as mushrooms have chemical structures similar to neurotransmitters and can act as agonists of receptor pathways involved in psychiatric conditions. Harnessing this activity as therapy for chronic mental health and pain diseases may offer benefits where therapeutic needs are currently unmet. Considering the high morbidity and mortality rates associated with these disorders and the economic burden placed on health systems, it is imperative competent treatment options are investigated. Bioactives such as psychedelics effect a person’s cognition, emotion and mood often having a long-lasting effect on symptoms of mental illness. Interest has arisen in tryptamine derivatives LSD and psilocybin and actives including hericenones and erinacines for the treatment of mood disorders, anxiety disorders, PTSD and addiction. Additionally, compounds acting as agonists of serotonin receptors may aid in alleviating symptoms of inflammatory conditions by regulating pro-inflammatory cytokines TNF and IL. While the potential benefits of such mycotherapy emerge, studies are warranted to determine their full pharmacokinetic and pharmacodynamic profiles before implementation as alternative drug therapy can be considered. While addiction and dependence do not appear to be an issue with such compounds, tolerance and cumulative effects must be considered. The complete aetiology of mental health disorders remains undetermined with the relationship between the mind, the brain and CNS a multifaceted interaction. As knowledge emerges giving a better understanding of mental illness, advances in neuropharmacology will undoubtedly follow. Perhaps such advances will include mycotherapy. Currently, the biggest challenge associated with psychedelic pharmacology studies is prohibition where psychedelics are classed as a Schedule 1 drug and the social stigma associated with their use. Chong et al., 2021 implied a predictive analysis method to investigate the molecular mechanisms of *H. erinaceus* [124], perhaps this offers a novel means of determining efficacy prior to clinical trials in line with prohibition. Other methods including ontology and Kyoto Encyclopedia of Genes and Genomes pathway analysis to investigate fungal biologics may also be implemented. Using animal species to model depression system in humans is currently standard protocol. Such assays allow researchers to investigate neural circuitry and molecular and cellular pathways in acute and chronic states. While such models off advantages, perhaps going forward inclusive studies of depression may employ several strains and multiple testing models. Where studies must compare genetic and epigenetic expressions, and activity to better extrapolate to human patients. Until a more comprehensive understanding of the aetiology of depression is established critical analysis of current drug therapy and fungal bioactives is warranted on existing animal and clinical studies to accurately determine the exact mode of action of fungal biologicals. Adopting ‘Quadruple Helix’ framework that unites academia, industry, policy, and society will further enable and support bioprospecting, development, testing and validation of bioactives of interest extracted from higher fungi (mushrooms) such as to advance the field of chronic pain and mental health disorders and conditions. Currently, international drug policies are slowly evolving as the potential benefits of these compounds are being revealed; however, care must be taken at clinical therapy stage.

## Figures and Tables

**Table 1:** Treatment of mental health disorders.

Disorder	Treatment	Mode of Action	Efficacy	Side Effects
Anxiety disorders *	SSRIs e.g., sertraline, escitalopram	Inhibit the reuptake of 5-HT	First-line treatments for PD, GAD, SAD, and PTSD [12]	GI problems (nausea, diarrhoea, dyspepsia, bleeding), dry mouth, headaches, dizziness, anxiety, insomnia, and sexual dysfunction [13]
	SNRIs e.g., venlafaxine, duloxetine	Inhibit the reuptake of NE and 5-HT (and/or DA)		
	TCAs e.g., amitriptyline, imipramine	Inhibit the reuptake of NE and 5-HT	Equivocal efficacy with SSRIs; however, cause more adverse side effects due to their anticholinergic activity [14]	Nausea, dry mouth, constipation, weight gain, blurred vision, light-headedness, confusion, sedation, urine retention and tachycardia [15]
	MAOIs e.g., moclobemide, phenelzine	Inhibit the mitochondrial enzyme monoamine oxidase	Third-line treatment for refractory SAD and PD, i.e., considered for patients who are non-responsive to other treatments [16]	Dry mouth, nausea, diarrhoea, constipation, drowsiness, insomnia, dizziness/or light-headedness, fatigue, urinary problems, sexual dysfunction, hypertensive crisis reaction, and serotonin syndrome [17]
	Benzodiazepines e.g., alprazolam, diazepam, clonazepam	Positive allosteric modulators of GABA-A, resulting in increased frequency of chloride channel opening	Effective and fast acting in the treatment of GAD. Recommended as second-line therapy and for short duration use due potential risks of tolerance, dependence, abuse, or misuse [13]	Drowsiness, lethargy, fatigue, and potential for dependence. Higher doses can cause impaired motor coordination, dizziness, vertigo, slurred speech, blurry vision, mood swings, euphoria and hostile or erratic behaviour
	Pregabalin	Calcium channel modulator [18]	Effective as a monotherapy for GAD, or as an adjunct to SSRIs/SNRIs in treatment-resistant GAD [19,20]	Sedation, dizziness, somnolence, dry mouth, amblyopia, diarrhoea, weight gain and potential for dependence [12]
	Buspirone	High affinity for 5-HT_1A_ receptors [21]		Nausea, headaches, dizziness, and fatigue [13]
Mood disorders *	Lithium	Multiple mechanisms including modulation of (GABA)-ergic and glutamatergic neurotransmission, and alteration of voltage-gated ion channels or intracellular signalling pathways [22,23]	First-line treatment for prevention of manic and depressive episodes of bipolar disorder (BD) [24]	Cardiac problems, cognitive problems, acne, psoriasis, thyroid problems, nausea, vomiting, weight gain, hyponatremia, sedation, decreased libido, and teratogenic [25]
	valproic acid		First-line treatment for acute mania and maintenance of BD [26]	Cardiac problems, cognitive problems, hair loss, hypothyroidism, aplastic anaemia, Leukopenia, increased transaminases, hepatitis, SLE-like syndrome, hyponatremia, tremor, decreased libido, infertility and teratogenic [25]
	Carbamazepine		Effective as a monotherapy to treat manic symptoms of bipolar or as adjunct to lithium or valproic acid [27]	Cardiac problems, cognitive problems, acne, hair loss, hypothyroidism, PCOS. diarrhoea, nausea, vomiting, pancreatitis, increased transaminases, metabolic syndrome, weight gain, sedation, tremor, decreased sexual function, infertility and teratogenic [25]
Psychotic disorders	First-generation antipsychotics (FGA) e.g., Chlorpromazine, haloperidol	D2 antagonists: work by inhibiting dopaminergic neurotransmission [28]	Effective in the treatment and maintenance of schizophrenia, acute mania with psychotic symptoms, major depressive order with psychotic features, and delusional disorder [28]	Adverse effects are drug specific and include anticholinergic effects (dry mouth, blurry vision, tachycardia, constipation), sedation, distonias, weight gain, increased lipids, parkinsonism (tremor, rigidity, bradykinesia), akathisia tardive dyskinesia, sialorrhea, orthostatic hypotension, neuroleptic malignant syndrome, sexual disfunction, neutropenia/agranulocytosis, and myocarditis [29]
	Second-generation antipsychotics (SGA) e.g., quetiapine, aripiprazole	Serotonin-dopamine antagonists: work by blocking D2 dopamine receptors as well as serotonin receptor antagonist action [28]	Same clinical efficacy as FGA, with the exception of clozapine, which has unique efficacy against treatment resistant schizophrenia [30]	
Eating disorder	Olanzapine (SGA)	Block dopaminergic (D1-4 antagonism) and serotonergic (5-HT_2A/2C_ antagonism) receptors [31]	Effective as an adjunctive therapy in treatment of AN, increasing appetite and decreasing anxiety and ruminating thoughts involving body image and food [32]	Dizziness, orthostatic hypotension, hypercholesterolemia, hypertriglyceridemia, hyperglycaemia, weight gain, extra-pyramidal symptoms, dry mouth, hyperprolactinemia, and insomnia [32]
	Antidepressants(SSRIs, SNRIs, TCAs, MAOIs)	Defined above	Effective as an adjunctive therapy in treatment of BN and BED, reducing the crisis of binge eating, purging phenomena and improving mood and anxiety [33]	Listed above
	Mood stabilizers e.g., topiramate	Blocks voltage gated sodium channels, enhances GABA-A receptor activity, reduces membrane depolarization by AMPA/Kainate receptors and is a weak inhibitor of carbonic anhydrase [34]	Shown efficacy in treatment of BN and BED, reducing the crisis of binge eating, purging phenomena and promoting weight loss (in overweight or obese patients) [33]	Paraesthesia, fatigue, cognitive problems, dizziness, somnolence, psychomotor slowing, memory/concentration difficulties, nervousness, confusion, weight loss [34]
Impulse control, addiction, and obsessive-compulsive disorders	Antidepressants e.g., SSRIs and clomipramine (TCA)	Potently inhibit the reuptake of 5-HT	Effective as a monotherapy or as an augmentation agent in the treatment of impulsive (PG, KM, TTM, IED and pyromania), addiction and compulsive disorders [35,36,37,38,39]	Listed above
	Mood stabilisers e.g., olanzapine, carbamazepine	Defined above		
	Naltrexone	Non-specific competitive opioid antagonist with highest affinity for the mu-opioid receptors in the CNS [39]		Nausea, vomiting, abdominal pain, decreased appetite, dizziness, lethargy, headaches and sleep disorders [40]
Personality disorders	Antidepressants (SSRIs, SNRIs)	Defined above	Shown efficacy in the treatment of BPD [41,42]	Listed above
	Quetiapine			
	Naltrexone			

Abbreviations: SSRI = selective serotonin reuptake inhibitors, SNRI = serotonin and norepinephrine reuptake inhibitor, TCA = Tricyclic antidepressants, MAOIs = Monoamine oxidase inhibitors, FGA = first generation antipsychotics, SGA = second generation antipsychotics, 5-HT = 5-hydroxytryptamine, NE = norepinephrine, DA = dopamine, GABA = γ-AMPA = aminobutyric acid, α-Amino-3-hydroxy-5-methyl-4-isoxazolepropionic acid, PD = panic disorder, GAD = generalised anxiety disorder, SAD = social anxiety disorder, PTSD = post-traumatic stress disorder, BD = bipolar disorder, AN = anorexia nervosa, BN = bulimia nervosa, BED = binge eating disorder, PG = pathological gambling, KM = kleptomania, TTM = trichotillomania, IED = intermittent explosive disorder, BPD = Borderline Personality Disorder, SLE = systemic lupus erythematosus, PCOS = Polycystic ovary syndrome, CNS = central nervous system. * Many drugs listed for the treatment of anxiety are also employed for the treatment of mood disorders including SSRIs, NSRIs and TCAs.

**Table 2:** Fungal biologics of therapeutic potential.

	Mescaline	Psilocybin/Psilocin	LSD
**Pharmacodynamics**	Naturally occurring substituted phenethylamine extracted from the peyote cactus	Naturally occurring indole-alkylamine (tryptamine) extracted from Psilocybe mushrooms	Semisynthetic indole-alkylamine (ergoline) derived from lysergic acid found in *Claviceps purpurea*
	5-HT releasing agent, catecholamine-like structure [77]	Close structural analogue of 5-HT	Close structural analogue of 5-HT
	Primarily interacts at 5-HT_2A/2C_ and α_2-_adrenergic receptors [78]	Primarily interacts at 5-HT_2A/2C_ and 5-HT_1A_, 5-HT_2C_ [79]	Mixed 5-HT_2_/5-HT_1_ receptor partial agonist [80]
	Low binding affinity at dopaminergic and histaminergic receptors [78]	Indirectly increases DA concentration but has no affinity for D2 receptors [81]	High affinity dopaminergic, adrenergic, and histaminergic receptors [82,83]
**Pharmacokinetics**	Can be ingested orally, smoked, or insufflated	Can be ingested orally or intravenously	Can be ingested orally, smoked, injected, or snorted
	Relatively low-potency: active doses in the 200−400 mg range) [84]	20× more potent than mescaline: active doses in 10–30 mg range [81]	2000× more potent mescaline: active doses in 25–200 μg range [85]
	Rapidly absorbed in GI and distributed to the kidneys and liver	Rapidly absorbed and dephosphorylated to psilocin (bioactive form) [81]	Rapidly absorbed in GI and distributed to tissues and organs
	Low lipid solubility, weak ability to penetrate BBB [77]	Lipid soluble, can easily cross BBB [86]	Can easily cross BBB [87]
	Detoxification via oxidative deamination	Detoxification via demethylation and oxidative deamination	Detoxification via N-dealkylation and/or oxidation processes
	Long-lasting, half-life of 6 hrs	Half-life of 3 hrs	Half-life of 3.6 hrs
	Eliminated in urine mainly in the unchanged form (81.4%) and the remaining as the metabolite TMPA [88]	Eliminated in urine mainly as glucuronidated metabolites (80%) as well as unaltered psilocybin (3–10%) [89]	Eliminated in urine mainly as metabolites, only 1% of the dose is excreted unchanged) [87]
**Efficacy**	Acute experiences of psychological insight during mescaline use are associated with self-reported improvements in anxiety disorders, depression, and substance abuse [78,84]	Therapeutic efficacy in treating mood and anxiety disorders, depression, cluster headaches, chronic pain, intractable phantom pain, obsessive-compulsive disorder, and substance abuse [79,81,90]	Therapeutic efficacy in treating anxiety disorders, depression, cluster headaches, obsessive-compulsive disorder, substance abuse, psychosomatic illnesses, and anxiety in relation to life-threatening diseases [91,92]
